# Open tibia plateau fracture with intra-osseous dislocation of the patella and quadriceps tendon rupture: a case report

**DOI:** 10.1186/s12893-020-00770-3

**Published:** 2020-05-25

**Authors:** Daohong Zhao, Weiqiang Li, Luping Liu, Ning Lu, Leijie Chen, Jun Zhang

**Affiliations:** Department of Orthopaedics, The Second Affiliated Hospital of Kunming Medical University, Kunming, China

**Keywords:** Tibial plateau fracture, Quadriceps tendon rupture, Knee dislocation

## Abstract

**Background:**

Both tibial plateau fractures and extensor apparatus injuries are serious injuries to the knee joint that generally do not occur in the same patient. We report a rare case of open tibial plateau fracture combined with quadriceps tendon rupture and complete displacement of the patella into the tibial plateau fracture.

**Case presentation:**

The case involved a male who was 19 years old who had been in a motorcycle accident. The patient was admitted to our department with an open tibial plateau fracture 3 h post-injury. X-ray showed a tibial plateau fracture and complete displacement of the patella into the tibial plateau. CT showed an avulsion fracture in the patella and tibial intercondylar eminence. Concomitant quadriceps tendon injury and both anterior and posterior cruciate ligament tibial insertion avulsion fractures were considered. The operative findings of emergency surgery confirmed our preoperative diagnosis. Single-stage quadriceps tendon repair and ORIF for the tibial plateau fracture were performed. Satisfactory restoration of function was acquired at the last follow up.

**Conclusion:**

The most difficult aspect of this case was the determination of the cause of the intra-osseous dislocation of the patella into the tibial plateau. The most likely mechanism of the injury may be that the patient experienced transient posterior dislocation of the knee during the injury. Rupture of the quadriceps tendon should be considered with posterior dislocation of the knee, and the patella was pushed into the tibial plateau fracture by force after the rupture of the quadriceps tendon.

## Background

Tibial plateau fracture and quadriceps tendon injury are severe injuries caused by violent trauma. Knee dislocation (KD) is a rare injury and can be accompanied by extensor apparatus injury, such as patellar ligament injury, patellar fracture, and quadriceps tendon rupture [1, 2]. Although a few cases of extensor apparatus injury combined with knee dislocation have been described in the literature [2], to the best of our knowledge, no cases of quadriceps tendon injury combined with tibial plateau fracture have been reported previously. The following might be the first such case.

## Case presentation

The patient was a 19-year-old male who had been in a motorcycle accident. The patient was admitted to our department as an open tibial plateau fracture 3 h post-injury. The emergency medical staff at the scene of the injury provided simple dressing and stabilization. The main complaint of the patient was pain and bleeding with limited movement of the knee.

**Fig. 1:**
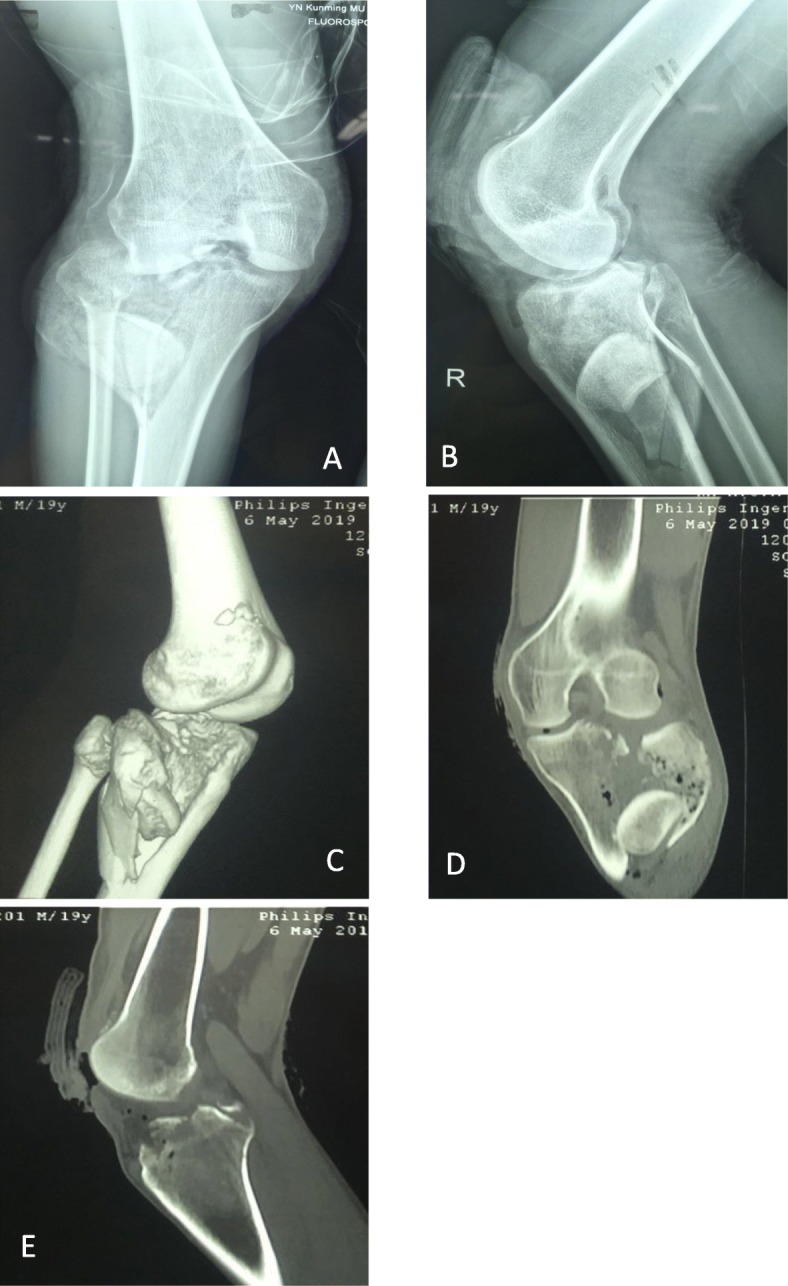
Preoperative radiographs: **a** AP view of radiograph; **b** lateral view of radiograph; **c** 3D CT scan; **d** coronal view of CT scan; **e** sagittal view of CT scan

**Fig. 2:**
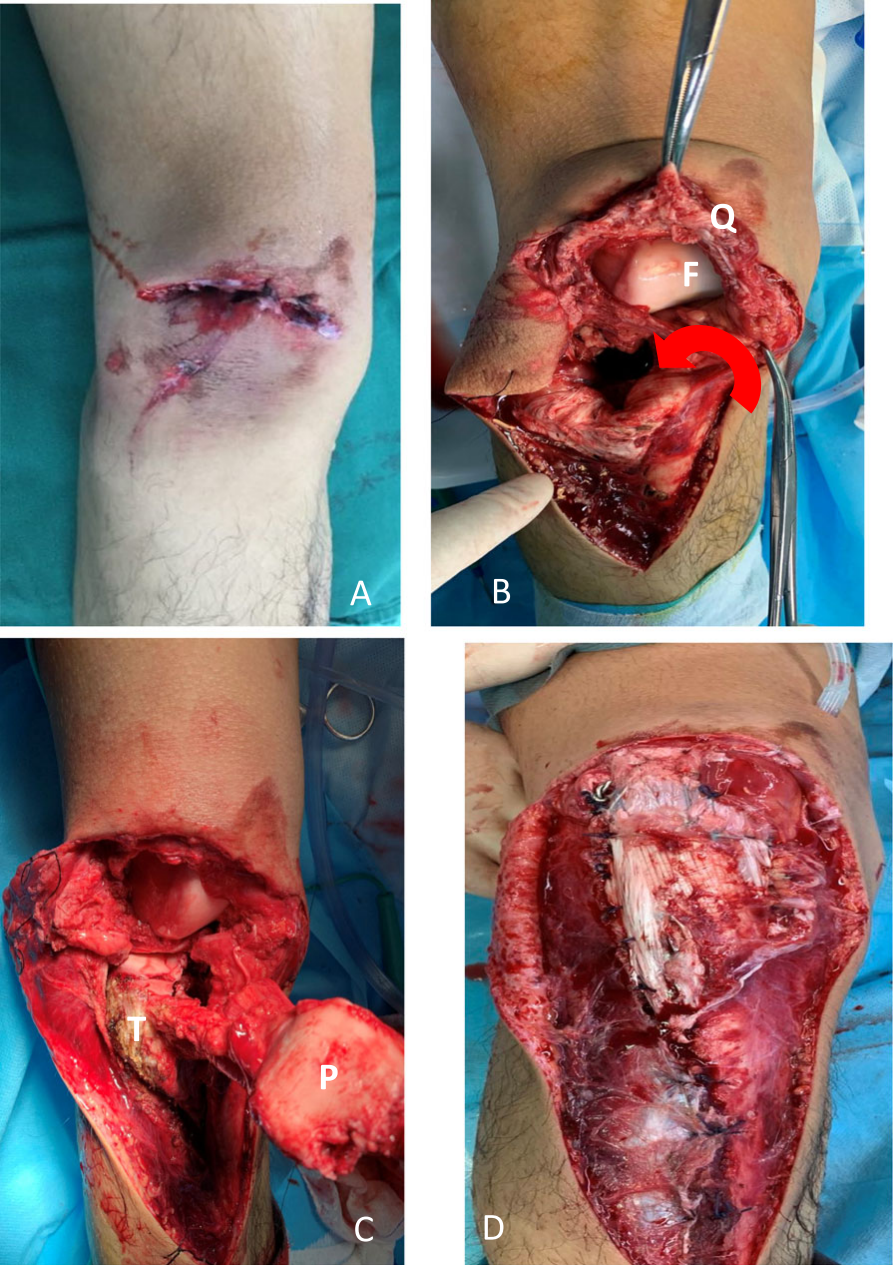
Intra-operative findings and procedures: **a** appearance of wound; **b** intra-operative findings; **c** step-by-step procedures; **d** appearance after tibial plateau fixation and quadriceps tendon repair; **F**: femoral; **P**: patella; **Q**: quadriceps; **T**: tibial plateau; shows the direction of the patella

On physical examination, after an advanced trauma life support protocol was performed in the emergency department, the patient’s blood pressure and pulse were within normal limits. There was a 5-cm-long wound without active bleeding on the anterior aspect of the knee joint (Fig. 2). The femoral condyle was exposed, the suprapatellar pouch was empty, and the injury endpoint was visible at the quadriceps tendon. Internal and external stress tests of the knee joint were negative, and anterior/posterior drawer tests were not able to be performed, as the patient could not tolerate them. The patient had a normal dorsalis pedis pulse without neurovascular injuries or any other accompanying injuries. Plain radiographs showed complete intra-osseous dislocation that locked into a tibial plateau fracture. Computed tomography showed a partial avulsion fracture of the patella and intercondylar eminence (Fig. 1).

The patient was taken to the operating room for emergency surgery. Intraoperatively, the patella was found to have rotated 360° and was completely locked into the tibial plateau fracture. The dislocated patella was found inside the tibia, approximately 10 cm below the tibia plateau. There were avulsion fractures of the anterior/posterior cruciate ligament insertions on the tibia and a partial patellar avulsion fracture with a “hat-off” type rupture of the quadriceps tendon at the attachment point on the patella; these operative findings confirmed our preoperative diagnosis. After thorough surgical debridement, the patella was pulled out. The tibial plateau fracture was restored and stabilized using an anatomical steel plate (Double Medical Technology, China). The quadriceps tendon was repaired with 3 anchors (Arthrex, USA) in the upper pole of the patella, and then a 2-mm transverse tunnel was established by using K-wire in the middle of the patella, and a 1-mm wire was guided to the patella and crossed the quadriceps tendon, leading to a tension band fixation (Fig. 2). Knee joint movement confirmed stable fixation, and the anterior/posterior drawer tests were positive (grade 2). As preoperative imaging did not show any apparent displacement of the tibial intercondylar eminence after the avulsion fracture, we performed no special procedure to manage it.

**Fig. 3:**
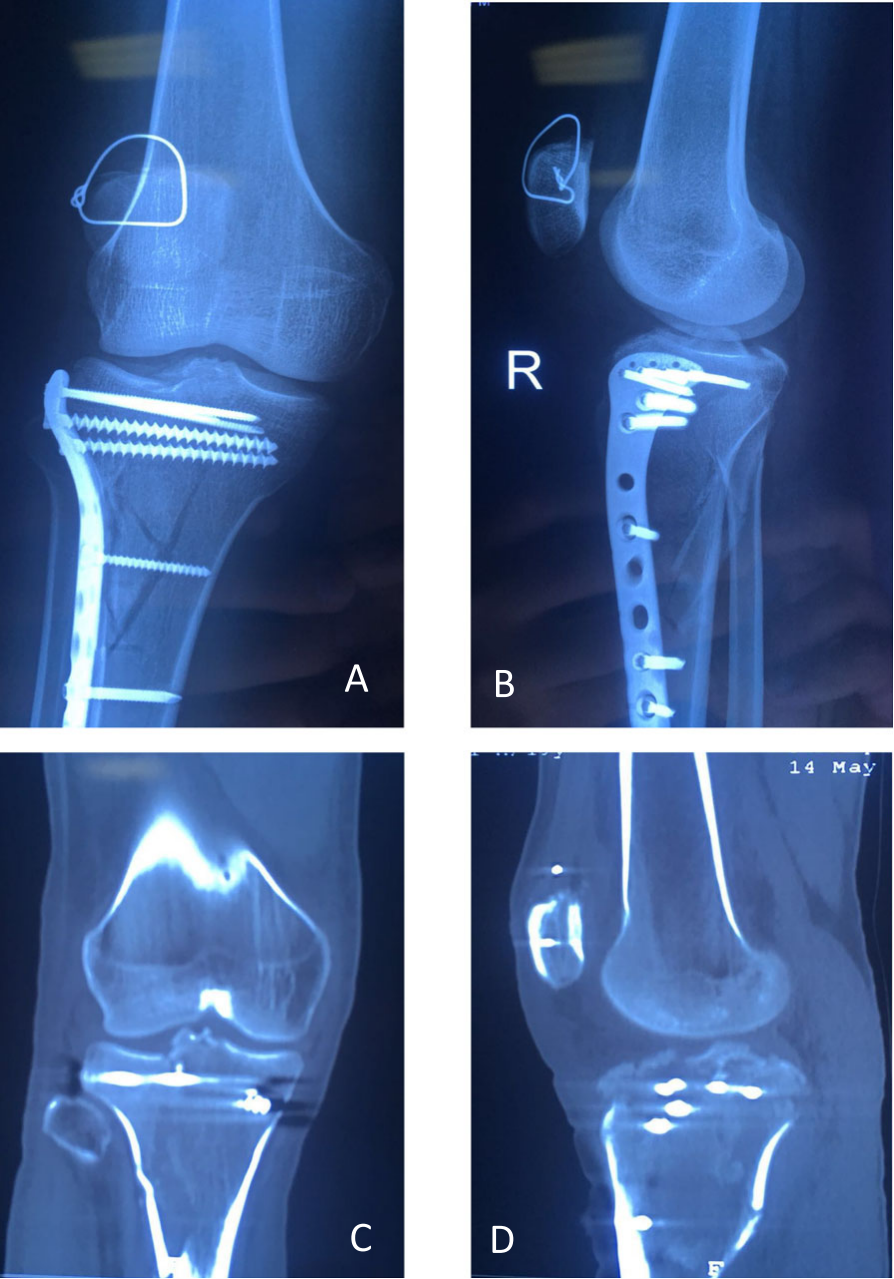
X-ray and CT in the 3rd postoperative follow-up: **a** AP view of radiograph; **b** lateral view of radiograph; **c** coronal view of CT scan; **d** sagittal view of CT scan

Postoperative wound healing was satisfactory, without infection or other complications. Postoperative imaging showed anatomical restoration of the tibial plateau fracture, with satisfactory positioning of the patella (Fig. 3). The patient started functional rehabilitation with the use of a brace at 3 weeks postoperatively. The early physiotherapy focused on gradually regaining ROM, including contiguous passive motion and quadriceps function with closed chain exercises. The knee range of motion was 0–110° at 6 weeks postoperatively. Partial weight-bearing exercise was planned to start at 6 weeks postoperatively, with wire removal at 8 weeks postoperatively. The patient returned at 3 months post-surgery; he was able to walk without a limp and gained full range of knee motion without any complaint of patellar or knee subluxation and was already back to normal life. A stable joint had been obtained, and the quadriceps tendon strength was grade 5/5. The patient’s knee function was evaluated subjectively using the IKDC 2000 knee-ligament standard evaluation form. The IKDC score of the injured knee was 85 at the final follow-up. Written informed consent for the case report was obtained from the patient and his family.

## Discussion and conclusions

In this manuscript, we describe a rare clinical case of open tibial plateau fracture with concomitant quadriceps tendon injury and complete patellar malposition into the tibial plateau, which has never been described in the literature previously. To date, this is the first case report on a tibia plateau fracture with intra-osseous dislocation of the patella. The hardest thing to understand was the cause of the intra-osseous dislocation of the patella into the tibial plateau to this case. Tibial plateau fracture generally occurs as a result of high-energy trauma in young patients. The tibial plateau fracture absorbs most of the energy; therefore, injuries to the ligaments surrounding the knee joint are rare [3]. The present patient had complete patellar malposition into the tibial plateau fracture, suggesting that an extensor apparatus injury had occurred. Preoperative imaging showed an avulsion fracture of the proximal patellar edge, indicating potential quadriceps tendon rupture, which was subsequently confirmed intraoperatively. The patella must have been subject to high energy to cause complete patellar malposition into the tibial plateau fracture. As the preoperative imaging also showed avulsion fracture of the anterior/posterior cruciate ligaments, the patient might have experienced transient posterior KD during the injury. Thus, we propose that the injury mechanism was as follows. The force directed into the anterior aspect of the knee caused high-energy trauma, resulting in avulsion fracture of the cruciate ligaments and complete instability of the knee joint. Therefore, the energy could not be fully absorbed, resulting in quadriceps tendon rupture and tibial plateau fracture. After the knee joint was completely dislocated, the patella was pushed into the tibial plateau fracture region. The knee joint then spontaneously relocated after the injury; the field emergency staff had repeatedly confirmed that there was no knee dislocation, and so no relocation procedure was performed. As the quadriceps tendon was completely ruptured, the patella lost its leverage effect and was then completely locked into the tibial plateau fracture. Of course, the case was an open injury; another possible mechanism was that a direct impact to the extensor apparatus causing the open wound led to the quadriceps rupture and malposition of the patella. However, it is difficult to explain how the patella was completely locked in the tibial plateau fracture. The most likely mechanism of the injury may be that the patient experienced transient posterior dislocation of the knee during the injury. Rupture of quadriceps tendon should be considered with posterior dislocation of the knee, and the patella was pushed into the tibial plateau fracture by force after the rupture of the quadriceps tendon.

KD is not common in clinical practice. KD mainly involves two or more groups of ligaments at the knee joint and may be accompanied by injuries to the structures surrounding the knee joint, with varying degrees of neurovascular injuries [4] or fractures. However, KD with concomitant tibial plateau fracture is not common. Most such cases involve small marginal fractures or compression fractures at the tibial plateau, such as an anterior compression fracture at the tibial plateau after KD. KD with concomitant extensor apparatus injury is rare, and such injuries combined with quadriceps tendon rupture have never been reported. Liu et al. [1] reported 15 cases of posterior KD associated with extensor apparatus rupture. Of those 15 cases, 13 presented with patellar fracture and patellar ligament injury, and two presented with avulsion fracture at the tibial attachment point, while no cases presented with quadriceps tendon rupture; similar findings were reported by Wissman [5]. Quadriceps tendon rupture is most commonly caused by trauma and most frequently occurs in young males. The mechanism includes instantaneous contraction of the quadriceps and passive stretching of the tendon beyond its limits, resulting in rupture [6]; older adults often have chronic diseases and tendinopathy, and therefore might be affected bilaterally.

## Conclusion

To the best of our knowledge, this is the first case report of an extremely rare case of open tibia plateau fracture with intra-osseous dislocation of the patella and quadriceps tendon rupture. The mechanism of the injury may be that the patient experienced transient posterior dislocation of the knee during the injury, and the patella was pushed into the tibial plateau fracture after the rupture of the quadriceps tendon. This case adds to the existing knowledge regarding the importance of understanding the injury mechanism and of examining the ligamentous structures during the diagnosis of knee injury.

## Data Availability

Data associated with this study are retained at a central repository at the Orthopaedic Department, the Second Affiliated Hospital of Kun Ming Medical University. If there are any questions, please contact the corresponding author.

## Abbreviations

KD: Knee dislocation
ORIF: Open reduction with internal fixation
